# Association of serum brain-derived neurotrophic factor and adiponectin with anxiety, depression, and stress in deaf adults: a cross-sectional study

**DOI:** 10.3389/fnbeh.2026.1955159

**Published:** 2026-10-07

**Authors:** Rand Alsharq, Anfal AL-Dalaeen, Eman Saleh, Nour Batarseh, Abeer Ahmad Bahathig

**Affiliations:** 1 Department of Clinical Nutrition and Dietetics, Faculty of Allied Medical Sciences, Applied Science Private University, Amman, Jordan; 2 Department of Community Health, College of Applied Medical Sciences, Northern Border University, Arar, Saudi Arabia

**Keywords:** adiponectin, anxiety, brain-derived neurotrophic factor, deaf, depression

## Abstract

Individuals with disabilities face practical and social challenges beyond those experienced by non-disabled individuals, which may increase vulnerability to mental health problems. However, few studies have examined biochemical correlates of psychological health in this population. This study investigated the associations of serum brain-derived neurotrophic factors (BDNF) and adiponectin with anxiety, depression, and stress among deaf adults. A cross-sectional study was conducted among 204 deaf adults aged 18–45 years. Anxiety and depression were assessed using the DASS-21 questionnaire. Body composition analyses were performed using bioelectrical impedance analysis, and serum BDNF and adiponectin levels were measured. Serum BDNF concentrations were inversely associated with anxiety (*r* = −0.632, *p* < 0.001), depression (*r* = −0.732, *p* < 0.001), and stress (*r* = −0.795, *p* < 0.001). Similarly, serum adiponectin concentrations showed significant inverse correlations with anxiety (*r* = −0.422, *p* < 0.001), depression (*r* = −0.344, *p* < 0.001), and stress (*r* = −0.446, *p* < 0.001). In multiple linear regression analyses adjusted for age, sex, and body mass index (BMI), BDNF remained an independent inverse predictor of anxiety (*β* = −0.48, *p* < 0.001), depression (*β* = −0.55, *p* < 0.001), and stress (*β* = −0.62, *p* < 0.001). Adiponectin also remained independently associated with anxiety (*β* = −0.19, *p* = 0.03) and stress (*β* = −0.21, *p* = 0.02). Lower serum BDNF and adiponectin concentrations were independently associated with higher levels of anxiety, depression, and stress among deaf adults. These findings suggest that BDNF, and to a lesser extent adiponectin, may represent potential biomarkers of psychological distress in this population.

## Introduction

Hearing impairment and deafness are major public health issues that affect social development and communication (Organization WH, 2025), educational achievement, overall quality of life, and create economic burdens (Marquete et al., 2022). Deaf individuals often encounter health literacy challenges due to substantial communication barriers, including social, cultural, and economic factors (Marquete et al., 2022) which may limit their access to healthcare (Perrodin-njoku et al., 2022). Collectively, these challenges contribute to increased psychosocial vulnerability and place deaf individuals at a substantially higher risk of adverse mental health outcomes compared with the general population (Emond et al., 2015). Previous research has consistently shown that deaf adults, particularly women and younger individuals, experience higher levels of anxiety, depression, and psychological distress than hearing individuals (Kushalnagar et al., 2019; Kvam et al., 2006) emphasizing the need to better understand the underlying mechanisms and to develop targeted preventive and therapeutic approaches.

Anxiety, depression, and stress are among the most common psychiatric comorbidities associated with hearing loss, with evidence suggesting that their prevalence and severity vary across the lifespan (Kushalnagar et al., 2019). Epidemiological studies have demonstrated that individuals with hearing impairment experience poorer psychological well-being, reduced self-esteem, greater social isolation, and increased emotional distress (Id et al., 2024; James et al., 2022). People with hearing impairment in low- and middle-income countries often have limited access to mental health services, greater social and emotional challenges, and face stigma related to hearing loss, which can worsen mental health compared to those without hearing loss (Naseribooriabadi and Sadoughi, 2017).

Recently, the role of neurotrophic factors and adipokines not only forces in metabolic regulation (Mukherjee et al., 2025). The Brain-derived neurotrophic factor (BDNF) has emerged as an important regulator of peripheral metabolism, influencing appetite control and energy expenditure (Huang et al., 2024), synaptic plasticity, learning, memory, and emotional regulation (Tongiorgi, 2008). It plays a central role in maintaining normal brain function and adaptive responses to stress (Sharma and Fulton, 2013). Chronic inflammation and prolonged psychological stress have been shown to reduce BDNF expression and circulating levels, resulting in impaired neuroplasticity and altered emotional regulation (Kilic et al., 2025; Zhang et al., 2025). Consequently, reduced BDNF concentrations have been consistently associated with anxiety disorders, major depressive disorder, and stress-related conditions (Zhang et al., 2025). Studies have demonstrated significantly lower BDNF concentrations in individuals with depression, particularly during acute episodes (Huded et al., 2025; Shkundin and Halaris, 2023).

Neurodevelopmental alterations in cortical morphology and hemispheric asymmetry may reflect underlying genetic, cellular, and maturational processes that predispose individuals to internalizing and externalizing psychopathology (Pisani et al., 2023). These structural vulnerabilities provide a complementary framework for understanding how altered neuroplasticity-related biomarkers, including BDNF, may relate to psychological distress (Alsharq et al., 2026).

In addition to BDNF, adiponectin has emerged as an important biomarker linking metabolic regulation, inflammation, and neuropsychiatric health (Khan et al., 2017). Adiponectin is an adipose tissue-derived cytokine with anti-inflammatory, insulin-sensitizing, and cardioprotective properties (Shaheer et al., 2025). Beyond its metabolic role, adiponectin contributes to neuroprotection and neuronal function through pathways involving inflammation, oxidative stress, and neurotrophic signaling, including interactions with BDNF (Forny-Germano et al., 2019; Genini et al., 2025). Lower serum adiponectin levels have been associated with anxiety, depression, chronic stress, and several neurodegenerative and psychiatric disorders (Gorska-Ciebiada and Ciebiada, 2024). Individuals with mood, anxiety, and stress-related disorders exhibit significantly reduced adiponectin concentrations compared with healthy controls, suggesting that chronic inflammation and metabolic dysregulation may contribute to its alteration (Chourpiliadis et al., 2024).

Although deaf individuals consistently demonstrate a higher burden of anxiety, depression, and psychological distress, the biological mechanisms underlying these associations remain insufficiently understood. Importantly, no previous study has simultaneously examined the independent associations of serum BDNF and adiponectin concentrations with anxiety, depression, and stress among deaf adults. This represents a critical knowledge gap, particularly given the additional challenges posed by social stigma, communication barriers, and limited access to culturally appropriate mental healthcare in this region. Therefore, the present study aimed to investigate the associations between serum BDNF and adiponectin levels and symptoms of anxiety, depression, and stress among deaf adults in Jordian adults.

## Methods

### Study design and setting

A cross-sectional study was conducted between September and December 2025 to examine associations between obesity, serum BDNF, and adiponectin among deaf adults in Amman, Jordan. Ethical approval was obtained from the Institutional Review Board of the Faculty of Allied Medical Sciences (Approval No.: IRB/AMS-2025-20), and all procedures adhered to the Declaration of Helsinki. Participants were fully informed about the study objectives, procedures, and voluntary participation, and digital informed consent was obtained using Jordanian Sign Language to ensure accessibility.

A total of 204 deaf adults aged 18–45 years were recruited through deaf community groups. Communication was conducted exclusively in sign language by trained personnel. Inclusion criteria required a clinical diagnosis of profound bilateral hearing loss and primary use of sign language. All participants had congenital deafness, defined as hearing loss present from birth. Exclusion criteria included chronic medical conditions affecting metabolic status, pregnancy or lactation, medications influencing weight or metabolism, recent bariatric surgery, unilateral hearing loss, or dual sensory impairments.

### Sample size

Sample size estimation was based on population data reported by the Higher Council for the Rights of Persons with Disabilities, which estimates approximately 20,000 deaf individuals in Amman. Using a confidence level of 95% and a 7% margin of error, the target sample size was calculated to be 150–200 participants. A total of 204 participants were enrolled.

### Sociodemographic data

Sociodemographic and lifestyle data were collected using a structured questionnaire, including age, sex, educational attainment, employment status, marital status, income level, smoking status, medical history, and medication use. All items were translated into Jordanian Sign Language by a certified researcher.

### Anthropometric and body composition assessment

Body weight and height were measured using standardized procedures, and body mass index (BMI) was calculated as weight (kg) divided by height squared (m^2^). Participants were classified according to World Health Organization BMI categories (World Health Organisation, 2004). Waist and hip circumferences were measured using a non-elastic tape, and waist-to-hip ratio (WHR) was calculated accordingly; WHR values ≥0.90 for men and ≥0.85 for women were considered indicative of central obesity and increased cardiometabolic risk (World Health Organisation (WHO), 2008). Body composition was assessed using bioelectrical impedance analysis (InBody 270), providing measures of body fat percentage, visceral fat level, lean mass, skeletal muscle mass, and total body water. Measurements were conducted in the morning, with participants instructed to refrain from eating or drinking for at least 2–3 h before assessment (Czartoryski et al., 2020).

### Psychological assessment (DASS-21)

Psychological distress was assessed using the Depression, Anxiety, and Stress Scale-21 (DASS-21), a validated self-report instrument consisting of 21 items distributed across three subscales: depression, anxiety, and stress (seven items each). Responses were recorded on a 4-point Likert scale ranging from 0 (“did not apply to me at all”) to 3 (“applied to me very much or most of the time”). Scores for each subscale were calculated by summing the corresponding items and multiplying the total by two, with severity categorized according to established cut-off values.

The DASS-21 was translated into Jordanian Sign Language and administered by a researcher certified by the Jordanian First Association of Sign Language Interpreters; however, the psychometric properties of this specific translated version have not yet been independently validated in deaf adults.

### Blood sample collection and biochemical analysis

Blood samples (5 mL) were collected by a certified nurse from participants. Samples were transported under cold conditions and centrifuged at 3,000×*g* for 10 min to separate serum. Serum aliquots were stored at −20 °C until analysis. Circulating adiponectin and BDNF concentrations were measured using commercially available enzyme-linked immunosorbent assay (ELISA) kits according to the manufacturers’ instructions: the Human Adiponectin ELISA Kit (GenoChem World SL, Spain; Cat. No. GW0068Hu) and the Human BDNF ELISA Kit (GenoChem World SL, Spain; Cat. No. GW0371Hu). Absorbance was measured using a BioTek ELISA plate reader (USA).

### Statistical analysis

Data were analyzed using SPSS version 25. Descriptive statistics were expressed as mean ± standard deviation (SD) for continuous variables and frequencies (percentages) for categorical variables. Independent samples *t*-tests and one-way analysis of variance (ANOVA) were used to compare continuous variables between groups, while chi-square tests were used to examine associations between categorical variables. Pearson’s correlation coefficient was used to assess relationships between continuous variables. Multiple linear regression analyses were performed to determine the independent associations between serum biomarkers (BDNF and adiponectin) and psychological outcomes (depression, anxiety, and stress), adjusting for age, sex, and BMI. Statistical significance was set at *p* < 0.05.

## Results

Table 1 presents the sociodemographic characteristics of the study participants. The sample was nearly evenly distributed by sex, with a slightly higher proportion of females (52.5%) than males (47.5%). Most participants were single (63.7%) and approximately half held a bachelor’s degree (51.0%). The majority were nonsmokers (78.9%), and most reported no chronic diseases (95.0%). Monthly income was predominantly in the lowest category (150–200 USD; 68.3%).

**Table 1:** Sociodemographic characteristics of deaf adults (*n* = 204).

Variable	Category	*n* (%)
Sex	Female	107 (52.5)
	Male	97 (47.5)
Marital status	Married	64 (31.4)
	Single	130 (63.7)
	Widowed	2 (1.0)
	Divorced	8 (3.9)
Education level	Primary school	12 (5.9)
	Middle school	49 (24.0)
	High school	32 (15.7)
	Bachelor’s degree	104 (51.0)
	Postgraduate degree	7 (3.4)
Smoking status	Non-smoker	161 (78.9)
	Smoker	43 (20.6)
Chronic disease	No	190 (95.0)
	Yes	14 (5.0)
Following special diet	No	169 (82.8)
	Yes	35 (17.2)
Monthly income (dollars)	150–200	141 (68.3)
	200–500	51 (24.9)
	600	12 (5.9)

Table 2 summarizes the anthropometric and body composition characteristics of the participants. The mean age of the total sample was 31.2 ± 11.8 years, with no significant difference between males (30.8 ± 11.5 years) and females (31.6 ± 12.1 years; *p* = 0.150). Males had significantly higher body weight than females (84.5 ± 9.8 vs. 76.0 ± 9.7 kg; *p* = 0.031), while females showed significantly higher body fat percentage (43.9 ± 11.7 vs. 30.5 ± 9.8%; *p* < 0.001). In contrast, males demonstrated higher fat-free mass (56.0 ± 8.5 vs. 45.2 ± 7.8 kg; *p* = 0.011) and skeletal muscle mass (34.8 ± 9.6 vs. 26.6 ± 8.9 kg; *p* = 0.021). Waist-to-hip ratio was higher in males than females, although this difference was not statistically significant (*p* = 0.151).

**Table 2:** Anthropometric and body composition characteristics of deaf adults (*n* = 204).

Variable	Total (mean ± SD)	Male (*n* = 97)	Female (*n* = 107)	*t*(df)	*p*-value
Age (years)	31.2 ± 11.8	30.8 ± 11.5	31.6 ± 12.1	−0.48 (202)	0.630
Weight (kg)	80.0 ± 10.4	84.5 ± 9.8	76.0 ± 9.7	6.22 (202)	**<0.001**
Body mass index (kg/m^2^)	28.2 ± 12.5	23.0 ± 10.2	32.9 ± 13.1	−5.98 (202)	**<0.001**
Body fat (%)	37.5 ± 12.5	30.5 ± 9.8	43.9 ± 11.7	−8.82 (202)	**<0.001**
Fat-free mass (kg)	50.3 ± 9.0	56.0 ± 8.5	45.2 ± 7.8	9.46 (202)	**<0.001**
Skeletal muscle mass (kg)	30.5 ± 10.8	34.8 ± 9.6	26.6 ± 8.9	6.33 (202)	**<0.001**
Waist–hip ratio	0.94 ± 0.06	0.96 ± 0.05	0.92 ± 0.06	5.14 (202)	**<0.001**

Values are presented as mean ± standard deviation (SD). Sex differences were assessed using independent-samples *t*-tests. *t*(df) indicates the *t*-statistic with corresponding degrees of freedom. Bold values indicate statistically significant differences at *p* < 0.05.

Table 3 presents the severity distribution of depression, anxiety, and stress among participants. For depression, the most frequent category was moderate (40.2%), followed by severe (19.1%), with no significant sex differences across categories (*p* > 0.05). For anxiety, moderate severity was most common (34.8%), and a significant sex difference was observed only in the very severe category, which was higher among females (*p* = 0.03), while other categories showed no significant differences. For stress, moderate severity was most prevalent (38.2%), and significant sex differences were observed in the moderate (*p* = 0.03), severe (*p* = 0.04), and very severe (*p* = 0.01) categories, with females more frequently represented in higher severity levels.

**Table 3:** Severity of depression, anxiety, and stress among deaf adults (*n* = 204).

Indicator	Category	Total *n* (%)	Male *n* (%)	Female *n* (%)	*p*-value
Depression	Normal	33 (16.2)	16 (16.5)	17 (15.9)	0.953
	Mild	37 (18.1)	19 (19.6)	18 (16.8)	
	Moderate	82 (40.2)	39 (40.2)	43 (40.2)	
	Severe	39 (19.1)	18 (18.6)	21 (19.6)	
	Very severe	13 (6.4)	5 (5.2)	8 (7.5)	
Anxiety	Normal	44 (21.6)	20 (20.6)	24 (22.4)	0.920
	Mild	25 (12.3)	12 (12.4)	13 (12.1)	
	Moderate	71 (34.8)	36 (37.1)	35 (32.7)	
	Severe	28 (13.7)	14 (14.4)	14 (13.1)	
	Very severe	36 (17.6)	15 (15.5)	21 (19.6)	
Stress	Normal	40 (19.6)	22 (22.7)	18 (16.8)	0.413
	Mild	36 (17.6)	20 (20.6)	16 (15.0)	0.413
	Moderate	78 (38.2)	36 (37.1)	42 (39.3)	0.413
	Severe	32 (15.7)	13 (13.4)	19 (17.8)	0.413
	Very severe	18 (8.9)	6 (6.2)	12 (11.2)	0.413

Bolded values indicate statistical significance at *p* < 0.05.

As shown in Figure 1, serum brain-derived neurotrophic factor (BDNF) was strongly and inversely correlated with anxiety (*r* = −0.632, *p* < 0.01), depression (*r* = −0.732, *p* < 0.01), and stress (*r* = −0.795, *p* < 0.01). Serum adiponectin also demonstrated significant inverse correlations with anxiety (*r* = −0.422, *p* < 0.01), depression (*r* = −0.344, *p* < 0.01), and stress (*r* = −0.446, *p* < 0.01), although these associations were weaker than those observed for BDNF. Multiple linear regression analyses adjusted for age, sex, and BMI confirmed that BDNF was an independent negative predictor of anxiety (*β* = −0.48, *p* < 0.001), depression (*β* = −0.55, *p* < 0.001), and stress (*β* = −0.62, *p* < 0.001), with the models explaining 36–42% of the variance (*R*^2^ = 0.36–0.42). Adiponectin remained independently associated with anxiety (*β* = −0.19, *p* = 0.03) and stress (*β* = −0.21, *p* = 0.02), whereas its association with depression was not statistically significant after adjustment (*β* = −0.12, *p* = 0.09).

**Figure 1:**
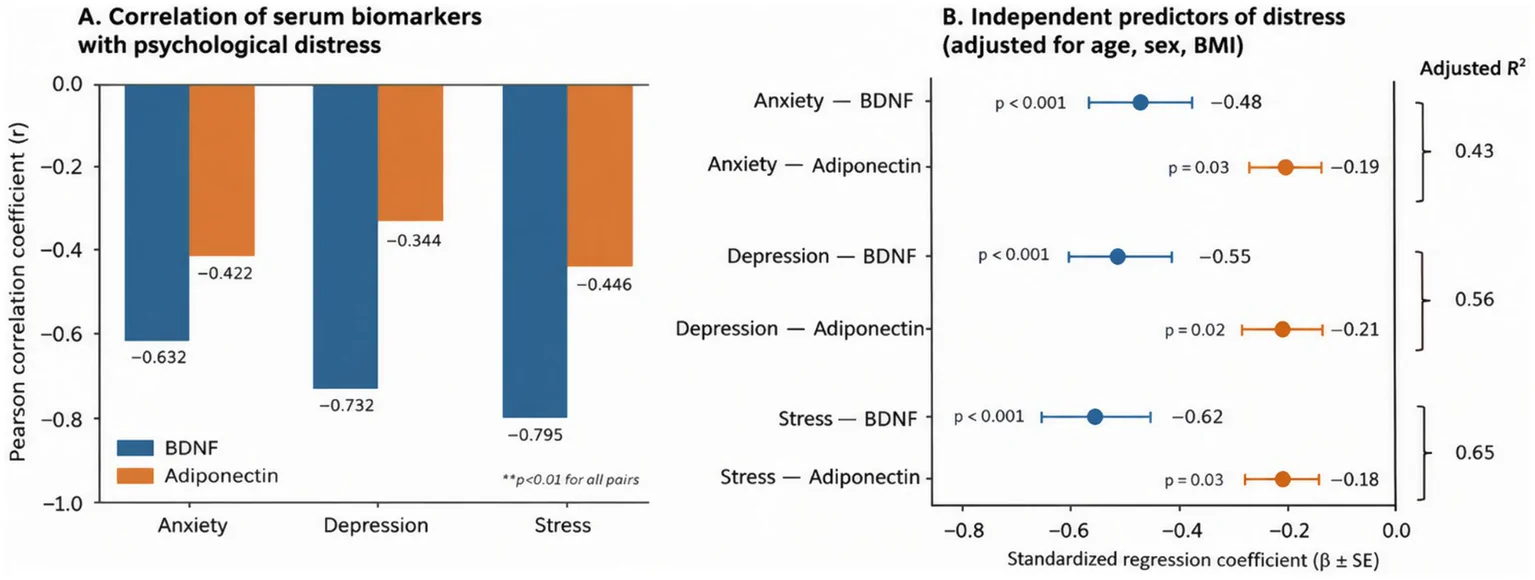
Association of serum BDNF and adiponectin with anxiety, depression, and stress among deaf adults (*n* = 204). **(A)** Pearson correlation coefficients (*r*) between each biomarker and psychological distress domain (all *p* < 0.01). **(B)** Standardized regression coefficients (*β* ± SE) from multiple linear regression models adjusted for age, sex, and BMI. BDNF; Brain-derived neurotrophic factors.

## Discussion

The deaf population is susceptible to a greater risk of psychological disorders such as depression, anxiety, and stress. Importantly, these disorders in combination with hearing loss will affect the overall psychological well-being, which consequently affects productivity, academic performance, social life, and quality of life in this population.

To the best of our knowledge, the current study is the first to investigate the relationship between psychological distress and BDNF and adiponectin levels in deaf adults in Jordan. This cross-sectional- study identified that The mean BMI of the study sample was 28.2 kg/m^2^, which falls within the overweight range.combined with high percentages of moderate-to-severe stress, anxiety, and depression levels. Furthermore, the findings suggested that low BDNF and adiponectin levels were strongly associated with psychological distress in the study sample. When adjusting the model for age, sex, and BMI, BDNF persisted as an independent negative predictor of all psychological outcomes, while adiponectin remained significantly associated with anxiety and stress.

The increased incidence of different psychological distress among the deaf population is a common health issue. The present study revealed that the percentage of deaf participants experiencing moderate stress, anxiety, and depression was high (38.2, 34.8, and 40.2%, respectively), a finding supported by previous studies conducted in six low- and middle-income countries and the USA (New York City), which reported that hearing loss was associated with high stress (Stubbs et al., 2018), anxiety, and depression levels (Zhang et al., 2023). A recent study demonstrated that after a complete adjusted multivariable regression model, a study participant with moderate hearing loss was 1.16 times significantly higher than a participant with normal hearing (Weinstein et al., 2025).

Notably, sex differences revealed that deaf females experienced significantly higher rates of very severe anxiety and elevated stress among moderate, severe, and very severe categories but not depression compared to males. This is consistent with the study conducted by Gomaa et al. (2014), which showed higher stress levels among females with hearing loss, while males were more affected by depression. However, our results showed no difference in depression levels among sexes. It is worth noting that, this results also consistent with the global epidemiological trend reveals that females consistently showed higher burden than males for anxiety and other psychological stress (Chen et al., 2026; Kushalnagar et al., 2019). The risk of these mood disorders may be amplified in deaf females who experience unique socioeconomic and social challenges. Interestingly, deaf females in our sample showed a significantly greater percentage of body fat (43.9%), which is above the normal acceptable range, which may partially explain the higher risk of psychological distress. Indeed, the relationship between being overweight or obese and stress, anxiety, and depression has been well-studied in the previous studies (Barnett et al., 2023; Eik-Nes et al., 2022).

The BDNF, a member of the neurotrophic factor family that is highly expressed in the central nervous system, has always been associated with the pathophysiology of psychiatric disorders such as depression and general anxiety disorder (Shkundin and Halaris, 2023). However, its value as a key biomarker for the diagnosis of these disorders is controversial. Our study revealed an independent inverse association between serum BDNF levels and all psychological measures. In addition, multiple linear regression models confirmed that after adjusting for age, sex, and BMI, BDNF was consistently the strongest negative predictor of anxiety, depression, and stress. This result may indicate that psychological stress associated with hearing loss may trigger neurological changes, leading to decreased BDNF levels and worsening symptoms of stress, anxiety, and depression in this population. In contrast, Pisani et al. (2023) showed that hearing loss and tinnitus may be combined with lower BDNF levels. In turn, this decline in BDNF levels may contribute to the development of psychological distress among deaf people. However, there is a lack of deafness-related studies in this context, with only one study conducted by Basso et al. (2022) assessing the level of BDNF in people with tinnitus after cognitive behavioral therapy. They found that baseline hair-BDNF level was associated with tinnitus-related distress (Basso et al., 2022) which is consistent with our findings.

Chronic psychological stress may disrupt mitochondrial homeostasis through impaired bioenergetics, oxidative stress, calcium imbalance, and inflammatory signaling, potentially contributing to neuronal dysfunction and reduced neuroplasticity (Basso et al., 2022). Emerging precision genome-engineering technologies may further enable targeted investigation and modulation of molecular pathways underlying such neurobiological alterations, although their therapeutic application in psychological disorders remains largely experimenta (Elias and Stern, 2026).

Similarly, serum adiponectin showed significant negative association with distress parameters. Adiponectin acts as an anti-inflammatory and insulin-sensitizing hormone, recent evidence considers it to have neuroprotective role against anxiety, stress, and depression (Genini et al., 2025). In our study, adiponectin levels independently predicted anxiety and stress. Interestingly, its relationship with depression was attenuated and lost statistical significance after adjusting for age, sex, and BMI. This attenuation suggests that the correlation between adiponectin levels and depressive symptoms is largely affected by demographic factors and body composition. This is inconsistent with the results of a study including healthy adults. A systematic review and meta-analysis concluded that adiponectin levels are reduced in many psychological problems, including chronic stress response, anxiety, mood disorders, and trauma/stressor-related disorders (Genini et al., 2025). In contrast to our results regarding depression, systematic reviews and meta-analyses have shown that adiponectin levels in depressed individuals are significantly lower than those in healthy individuals (Lehto et al., 2010). In contrast, another systematic review showed that adiponectin levels are not affected by depression (Carvalho et al., 2014). However, the body of literature does not provide sufficient evidence on adiponectin levels in deaf people.

In summary, our findings reveal, for the first time, that BDNF and adiponectin act as possible biomarkers of psychological distress in the deaf population. However, this study has several limitations that must be acknowledged. First, due to the cross-sectional design, we cannot conclude the causal direction between declining biomarker levels and psychological distress. Second, serum biomarker levels may fluctuate due to unmeasured lifestyle factors, such as physical activity levels and specific dietary macro- and micro-components. Additionally, this study has many strengths, including being one of the first to assess dual biochemical markers (BDNF and adiponectin) alongside psychological distress exclusively in a deaf adult population. Furthermore, the use of the DASS-21, which was culturally and linguistically adapted into Jordanian Sign Language, ensured data validity. Several limitations should be acknowledged. The cross-sectional design precludes causal inference regarding the directionality of the observed associations. In addition, physical activity was not assessed in this study and may have confounded the observed BDNF associations. Exercise can acutely increase circulating BDNF and, with repeated training, may enhance BDNF-related neuroplasticity through lactate signaling and the PGC-1α/FNDC5 pathway. Therefore, variation in recent or habitual exercise could have affected serum BDNF levels and the regression estimates for anxiety, depression, and stress.

## Conclusion

This study demonstrated that deaf adults in Jordan experience a high prevalence of psychological distress, particularly moderate-to-severe symptoms of anxiety, depression, and stress. Lower serum concentrations of brain-derived neurotrophic factors (BDNF) and adiponectin were significantly associated with greater psychological distress, with BDNF emerging as an independent predictor of anxiety, depression, and stress Adiponectin remained independently associated with anxiety and stress but not depression. These findings suggest that impaired neuroplasticity and inflammatory pathways may contribute to the psychological burden experienced by deaf adults. Integrating biological markers with culturally appropriate psychological assessment may improve the identification of individuals at risk and support the development of targeted mental health interventions. Future prospective longitudinal studies are required to identify the precise causal trajectories and to evaluate whether targeted lifestyle interventions, such as tailored nutrition or exercise programs aimed at boosting BDNF and adiponectin, can alleviate the psychological burden in this population.

## Data Availability

The original contributions presented in the study are included in the article/supplementary material, further inquiries can be directed to the corresponding author/s.
